# Comparing the Efficacy of Angiotensin Converting Enzyme Inhibitors with Calcium Channel Blockers on the Treatment of Diabetic Nephropathy: A Meta-Analysis

**Published:** 2019-02

**Authors:** Zhaowei ZHANG, Chunlin CHEN, Shiwen LV, Yalan ZHU, Tianzi FANG

**Affiliations:** 1. Department of Pharmacy, Jin Hua Municipal Central Hospital, Jin Hua 32100, China; 2. College of Chemistry and Bio-Engineering, Yi Chun University, Yi Chun 336000, China

**Keywords:** Diabetic nephropathy, Angiotensin converting enzyme inhibitors, Calcium channel blockers, Meta-analysis

## Abstract

**Background:**

The angiotensin-converting enzyme inhibitors (ACEIs) could improve the symptoms of diabetic nephropathy. Whether the calcium channel blockers (CCBs) could be as effective as ACEIs on treating diabetic nephropathy is controversial. Here, we aimed to compare the efficacy of ACEIs with CCBs on the treatment of diabetic nephropathy by performing a meta-analysis of randomized controlled trials (RCTs).

**Methods:**

The Pubmed, Medline, Embase and The Cochrane Database were searched up to July 2017 for eligible randomized clinical trials studies. Effect sizes were summarized as mean difference (MD) or standardized mean difference (SMD) with 95% confidence intervals (*P*-value<0.05).

**Results:**

Seven RCTs involving 430 participants comparing ACEIs with CCBs were included. No benefit was seen in comparative group of ACEIs on systolic blood pressure (SBP) (MD=1.05 mmHg; 95% CI: −0.97 to 3.08, *P*=0.31), diastolic blood pressure (DBP) (MD= −0.34 mmHg; 95% CI: −1.2 to 0.51, *P*=0.43), urinary albumin excretion rates (UAER) (MD=1.91μg/min; 95% CI: −10.3 to 14.12, *P*=0.76), 24-h urine protein (24-UP) (SMD=−0.26; 95%CI: −0.55 to 0.03, *P*=0.08), glomerular filtration rate (GFR) (SMD=0.01; 95% CI: −0.38 to 0.41, *P*=0.95). On safety aspect, the risk of adverse reactions between ACEIs group and CCBs group are similar (RR=1.18; 95% CI: 0.61 to 2.28; *P*=0.61).

**Conclusion:**

Both ACEIs and CCBs could improve the BP, UAER, 24h-UP, and GFR of diabetic nephropathy to a similar extent.

## Introduction

Diabetic nephropathy (DN) is a major complication of diabetes. It is the main common cause of end-stage kidney disease (ESKD) (1). DN often develops with macrovascular disease including cardiovascular, cerebrovascular, and peripheral arterial diseases with a higher risk of morbidity and mortality (2). The mechanism of DN is very complex due to inflammation, hemodynamic effects and genetic predispositions which manifest in fibrotic lesion and eventually irreversible organ damage (3). Microalbuminuria is generally considered the earliest marker for the development of diabetic nephropathy (4). Some patients with microalbuminuria will progress to macroalbuminuria and eventually to ESRD (5). Moreover, Glomerular filtration rate (GFR), a critical index for assessment of renal dysfunction, starts to mildly decreases during the phase of microalbuminuria (6). As the increasing number of DN individuals, the development of cost-effective therapeutic strategies for these individuals is a crucial public health concern (7). The treatment of patients with DN can be divided into 4 major arenas: cardiovascular risk reduction, glycemic control, blood pressure control and inhibition of the RAS (8).

Angiotensin-converting enzyme inhibitors (ACEIs) is one type of antihypertensive drugs. It lower intraglomerular pressure via inhibiting renin angiotensin aldosterone system (RAAS). In addition, ACEIs has superior cardioprotective effects compared with other antihypertensive drugs (6). Therefore, ACEIs have been considered as the standard care in patients with diabetic nephropathy recommended by The American Diabetic Association (9). Calcium channel blockers (CCBs) could decrease albuminuria in patients with incipient diabetic nephropathy, but there are no guidelines recommended that CCBs is the standard care in treating DN (10–12).

Less is known about whether the ACEIs or CCB could be better to patients with DN. Therefore, the purpose of this meta-analysis was to compare ACEIs with CCBs on reducing albuminuria and improving GFR of patients with DN.

## Methods

### Search Strategy

We conducted search of PubMed, Medline, EM-BASE and COCHRANE (from Jan 1992 to Jul 2017) to identify all trials published in English involving the following search terms:(“Angiotensin Converting Enzyme Inhibitors”)(Mesh) OR alacepril OR benazepril OR captopril OR ceronapril OR cilazapril OR enalapril OR fosinopril OR perindopril OR ramipril OR delapril OR imidapril OR moexipril OR spirapril OR rentiapril OR trandolapril OR zofenopril OR libenzapril OR quinapril OR lisinopril AND (“Calcium Channel Blockers”)(Mesh) OR amlodipine) OR amrinone) OR anipamil OR benidipine OR bepridil OR berbamine OR cinnarizine OR diltiazem OR devapamil OR darodipine) OR dotarizine) OR efonidipine) OR emopamil OR felodipine OR fendiline OR flunarizine OR gallopamil OR isradipine) OR lacidipine OR lercanidipine OR lidoflazine OR lomerizine OR manidipine OR mepirodipine OR mibefradil OR monatepil OR nicardipine OR nifedipine OR nilvadipine OR nimodipine OR nisoldipine OR nitrendipine OR pranidipine OR prenylamine OR sesamoid OR verapamil OR oxodipine OR perhexiline AND nephropathies OR diabetic nephropathy OR diabetic kidney disease AND random) OR randomized OR randomised OR double blind OR placebo OR controlled OR randomized controlled trial.The protocol with details for this meta-analysis was published on the PROSPERO website (http://www.crd.york.ac.uk/PROSPERO/), (PROSPERO CRD 42016048199) ahead of the initiation of the literature search.

### Study Selection

Two investigators selected the studies independently and resolved any conflicts through discussion. The inclusion criteria for this meta-analysis were as follows: 1) study design: controlled or parallel RCT designs 2) population: patients with hypertension, diabetes and microalbuminuria or macroalbuminuria. The systolic blood pressure (SBP) ≥140 mmHg or diastolic BP (DBP)≥90 mmHg at baseline; microalbuminuria was defined as urinary albumin excretion rate (UAER) of 20–200 μg/min or 24-h urine protein (24h-UP) 30–300 mg. macroalbuminuria was defined as UAER≥ 200 μg/min or 24h-UP≥300 mg for the same specimens) 3) study treatment: studies comparing ACEIs treatment with CCBs treatment and having the outcomes of microalbuminuria or macroalbuminuria. The exclusion criteria were as follows: 1) studies failing to report the mean value and SD of the primary efficacy outcome 2) studies including patients with kidney disease secondary to causes other than diabetes 3) studies including patients with an active kidney transplant, undergoing kidney transplantation or undergoing dialysis.

### Data Extraction

Two investigators were responsible for extracting the data independently from each included RCT: first author’s name, year of publication, mean age of participants, type of diabetes among participants, definition of microalbuminuria, BP categories of participants, intervention(s) prescribed (with dosage levels), follow-up period (in months), the outcome of SBP, DBP, UAER, 24h-UP, GFR and adverse events. The main outcomes given with median and range were estimated as the Mean and Standard Deviation (13).

### Quality Assessment

The quality of the studies was evaluated independently by two members of our team as described in the Cochrane Handbook for Systematic Reviews of Interventions. We assigned values of low, unclear or high risk of bias to the following domains: random sequence generation, allocation concealment, blinding of participants and personnel, blinding of outcome assessment, incomplete outcome data, selective reporting and other biases. Disagreements were resolved by consensus.

### Outcome

Our team extracted data on the following outcomes: 1) primary outcomes: urinary protein measured as UAER (μg/min) and 24-h UP (mg/24 h); 2) secondary outcomes: renal function measured as GFR (ml/min/1.73m^2^); 3) adverse events (AEs).

### Sensitivity Analyses

Sensitivity analyses were conducted by excluding low-quality studies based on descriptions of randomization, allocation concealment, blinded assessment of outcomes to compare the change of results.

### Statistical analysis

For direct meta-analysis, the intervention of interest was ACEIs versus CCB therapy. The Q test and I^2^ statisticwere used to assess the presence and degree of heterogeneity. If heterogeneity was present or I^2^>30%, the random effect model was applied, otherwise, the fixed-effects model was used. Dichotomous data were presented as risk ratios (RRs), with 95% confidence intervals (Adverse effects). Weighted mean difference (WMD) was used for continuous outcomes (including SBP and DBP, GFR, UAER) along with their corresponding 95% confidence intervals (CIs).

All statistical analyses were performed using Review Manager (RevMan), version 5.3 (Cochrane Collaboration, Oxford, UK). *P*<0.05 was considered statistically significant, except for the test of heterogeneity where *P*<0.1 was used. These values were captured as the mean change from baseline to follow-up (with mean ±SD). The mean changes were calculated by subtracting the baseline values from the final values. Additionally, the standard deviations of the mean changes (SD(C)) were calculated according to the follow- formula: SD(C) =√SD(A)^2^+ SD(B)^2^− (2 × R × SD(A) × SD(B)) We assumed a pre-post study correlation R of 0.5 to get an estimate of the mean change in SD(C).

### Ethical approval

This article does not contain any studies with human participants or animals performed by any of the authors.

## Results

### Study selection

Overall, 686 papers were retrieved, of which198 duplicate papers were excluded. 467 papers were subsequently excluded based on review of title and abstract. Eight papers were subsequently excluded based on full-text review according to the inclusion and exclusion criteria. Six papers could not get data for extraction mean and SD. Seven papers with a total of 430 patients were included in this meta-analysis (Table 1).

**Table 1:** Characteristics of trials included in this meta-analysis

***Studies***	***N(T/C)***	***Age(yr)***	***Diabetic type***	***Albuminuria definition***	***Main intervention***	***Follow up (months)***
R.Romero^15^ (1992)	20 (10/10)	55.4±8.6	type2	24h-UP>500mg	catopril:60±17.5 mg/day; nifedipine:40 mg /day	6
Kirsten Nørgaard^16^ (1993)	15 (7/8)	T:43±6 C:42±8	type1	24h-UP>300mg	Spirapril:6mg/day + fursemide; Isradipine:5mg/day+ fursemide	6
Raffaele De Cesaris^17^ (1996)	46 (24/22)	T:54±5.7 C:56.0± 4.8	type 1 type2	UAER:30–300 μg/min	Benazepril: 10mg/day; Nicardipine: 20mg/day	6
Mario Velussi^18^(1996)	18 (9/9)	T: 55±2 C:56±4	type2	UAER:20–200 μg/min	Cilazapril:2.5–5 mg/day+thiazide or furoSemide; Amlodipine:5–10 mg/day+thiazide or furosemide	36
B L Salako^19^ (2002)	30 (15/15)	T:58.7±5.7 C:58.5±8.4	type2	24h-UP>300mg	Lisinopril5–40 mg/day+furosemide; Lacidipine2–8 mg/day+furosemide	3
M.Dalla Vestra^20^ 2004	180 (89/91)	T: 60±7 C: 58±7	type2	UAER:20–200 μg/min	ramipril:5–10 mg/day lercanidipine:10–20 mg/day	13
Roberto Fogari ^21^ 2005	121 (61/60)	T: 59.9±7 C:60.6±6	type2	24h-UP:30–300mg	lisinopril:10–20 mg/day; manidipine:10–20 mg/day	24

Abbreviations: T = ACEI/ARB group; C = CCB group; UAER = urinary albumin excretion rate; 24h-UP = 24h- urine protein

### Quality assessment

The risk of bias in each study was assessed using the criteria recommended by the Cochrane Handbook for Systematic Review of Interventions. Only one trial reported random sequence generation (14). One trial reported the method to generate the allocation sequence (15) (Fig. 1A and B).

**Fig. 1A:**
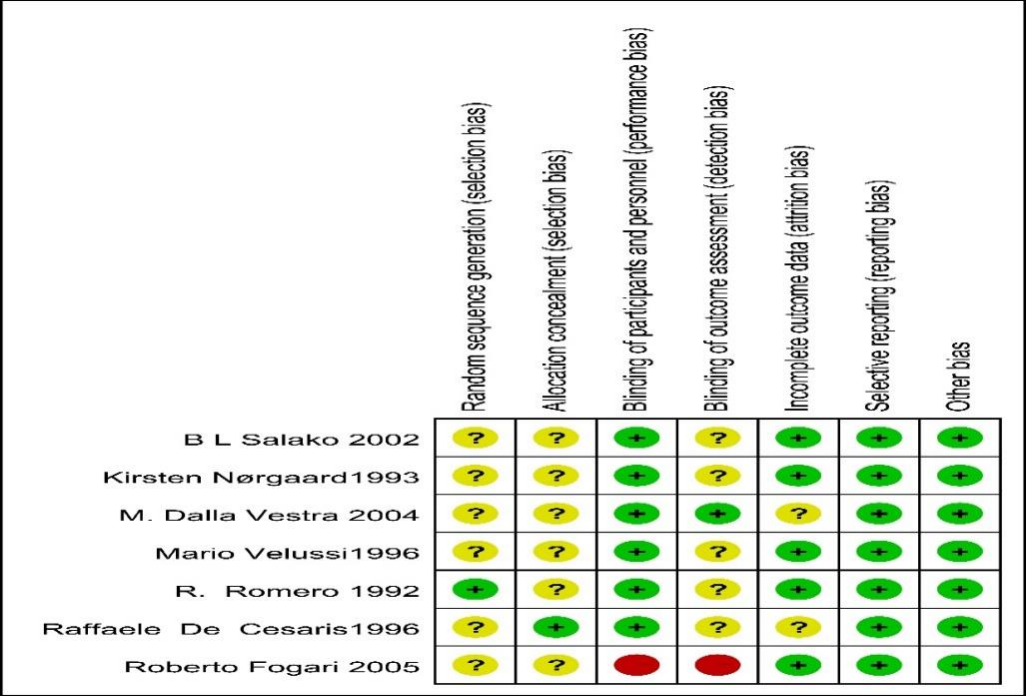
Risk-of-Bias Summary

**Fig. 1B:**
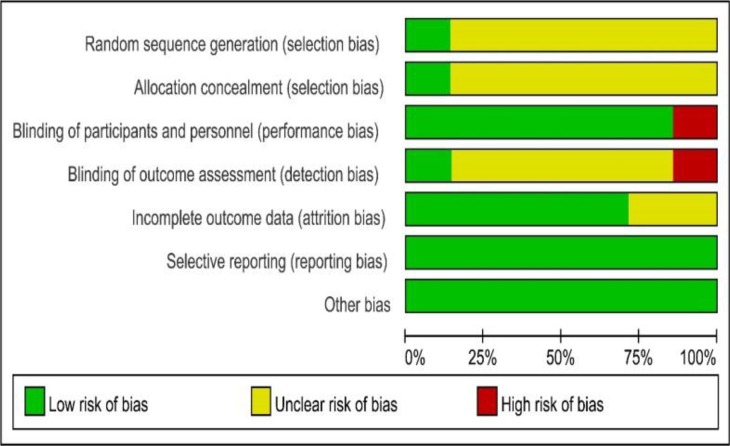
Risk-of-Bias Graph of bias

### Systolic blood pressure and diastolic blood pressure

Seven studies were included that compared the effect of ACEIs with CCBs on changing the SBP and DBP. There were no differences in the degree of change of SBP (MD=1.05 mmHg; 95% CI: −0.97 to 3.08; *P*=0.31). The treatment effects were homogeneous (*P*=0.85, I^2^=0%). Meanwhile, there were no differences in the degree of change of DBP (MD= −0.34 mmHg; 95% CI: −1.2 to 0.51; *P*=0.43). There was no significant heterogeneity among the seven studies (P=0.50, I^2^=0%).

### 24h-Urinary protein or Urine albumin excretion rate

**Fig. 2:**
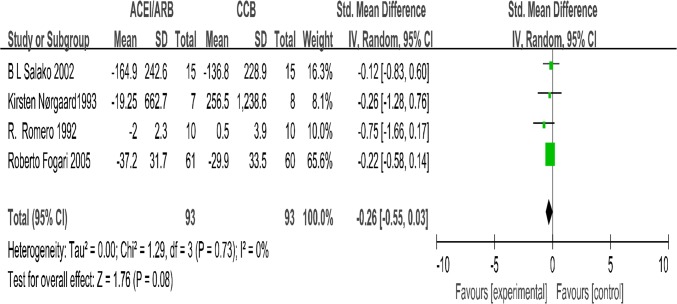
The changes in 24h-urine protein (24h-UP): Forest Plot of Comparison ACEI/ARB Versus CCB Only

**Fig. 3:**
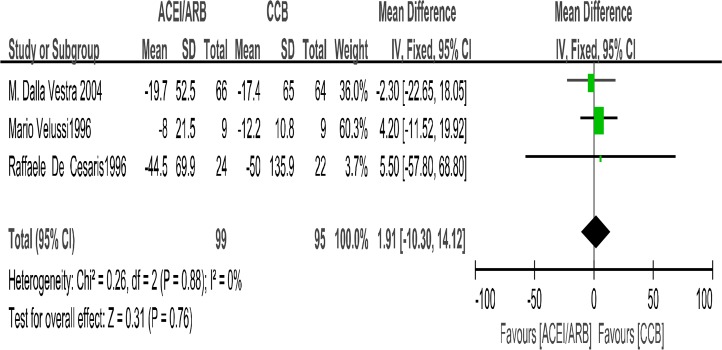
The changes in urinary albumin excretion rate (UAER): Forest Plot of Comparison ACEI/ARB Versus CCB Only

Four trials (14, 16–18) in our analysis compared the effect of ACEIs with CCBs on reducing 24-hour urinary protein with CCBs. The meta-analysis suggested that there were no differences in the degree of change of 24h-proteinuria levels (SMD= −0.26; 95% CI: −0.55 to 0.03; *P*=0.08, Fig. 2). The test for heterogeneity was low (*P*=0.73, I^2^=0%). Three papers (15, 19, 20) reported urine albumin excretion rate. The change in urine albumin excretion rate related outcome was not significantly different between the two treatment arms (MD=1.91μg/min; 95% CI: −10.3 to 14.12; *P*=0.76, Fig. 3). We recorded no significant interstudy heterogeneity among the 3 studies (*P*=0.88, I^2^=0%).

### Glomerular filtration rate

**Fig. 4:**
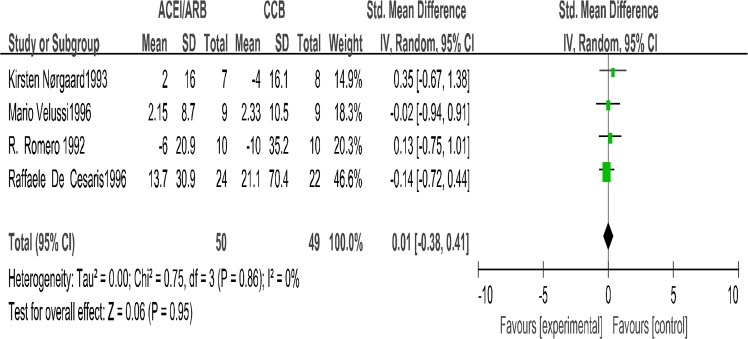
The changes in glomerular filtration rate (GFR): Forest Plot of Comparison ACEI/ARB Versus CCB Only

Four trials (14–16, 19) investigate the GFR for a total of 99 participants. There were no differences in the degree of change of GFR between ACEIs group and CCBs group (SMD=0.01; 95% CI: −0.38 to 0.41; *P*=0.95, Fig. 4). Heterogeneity between the 4 studies was not significant (*P*=0.95, I^2^= 0%).

### Primary safety endpoint: adverse events

**Fig. 5:**
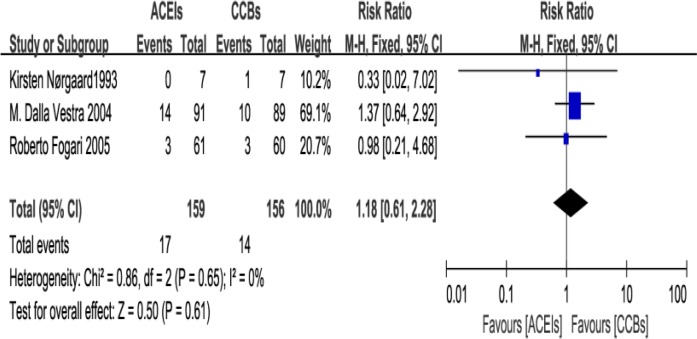
The changes in adverse reaction (ADR): Forest Plot of Comparison ACEI/ARB Versus CCB Only

Three papers (16,18,20) report the adverse events. A fixed effects model was used to compile the results because heterogeneity was small among the 4 studies (I^2^=0).There was no significant difference in the risk of adverse reactions between ACEIs group and CCBs group (RR=1.18; 95% CI: 0.61 to 2.28; *P*=0.61, Fig. 5).

### Sensitivity analysis

Removal of unblinded, low-quality studies from our analysis did not alter the results for the primary endpoint significantly.

## Discussion

Diabetic nephropathy (DN), a common complication in patients with diabetic, is characterized by hypertension, macroalbuminuria, microalbuminuria and abnormal renal function (21). The pathophysiological mechanisms in the development of DN are very complex. They may include glomerular hyperfiltration, glomerular and tubular epithelial hypertrophy, microalbuminuria. Activation of rennin-angiotensin system is involved in almost all the steps in the development of DN (22, 23). Drugs acting on the Renin-Angiotensin-Aldosterone System (RAAS) play a crucial role in the therapeutic regimen to prevent DN (24). Calcium channel blockers (CCBs) are recommended to add to the therapeutic regimen (10). It may act predominantly by relaxing the afferent glomerular arteriole, increasing intraglomerular pressure. ACEIs and CCBs have similar beneficial effects on reducing the progression of diabetic nephropathy (25,26). However, ACEIs have better effects on reducing the progression of diabetic nephropathy (27). This meta-analysis tries to further compare the effectiveness and safety of ACEIs with CCBs on treating diabetic nephropathy.

Our meta-analysis showed that ACEIs are not superior to CCBs in reducing SBP, DBP, GFR, UAER, and 24-h UP in DN patient with hyper-tension. Regarding safety, this meta-analysis showed that ACEIs group and CCBs group have similar incidence of adverse events. Vejakama (28) suggests a consistent reno-protective effect of ACEIs and angiotensin II receptor blocker (ARBs) over other antihypertensive drug in type 2 diabetes on the risk ratio of the outcome of albuminuria regression, microvascular complications, serum creatinine doubling, macroalbuminuria, microalbuminuria and ESKD but not the change of UAER and 24-h UP which are the main screening indicators of DN. Our result was the first time to compare ACEIs with CCBs in changing the UAER and 24-h UP. The different results of the two studies may be due to different observed indicator, baseline of BP and treatment duration. Lisinopril reduced albuminuria, but also GFR, to a greater extent than nisoldipine did in hypertensive IDDM patients with diabetic nephropathy during the first year of treatment (30). ACEIs and CCBs equally reduce the progression of nephropathy in hypertensive type 2 diabetics (26,29,30). It was consistent with our results, but these studies are not included in our meta-analysis due to unreported urinary albumin excretion or lack of the mean and SD of urinary albumin excretion.

There are some limitations to our study. Firstly, the studies included in our meta-analysis are relatively small, so do the population. Secondly, the methodological quality of the included studies was not very high. The methods of randomization were not clear in all the trials. Thirdly, drug effectiveness may vary with different follow up duration and different diabetic types, a subgroup analysis according to follow up duration and diabetic types would provide valuable insight. Unfortunately, our analysis was not possible with enough available data. Lastly, the CCBs are all calcium channel blocker of dihydropyridine, whether other kinds of CCBs have the same function remains unknown.

## Conclusion

Patients with hypertension and diabetic nephropathy derive similar benefit from ACEIs therapy and CCBs therapy on reducing BP, GFR, UAER and 24h-UP. The incidences of adverse reaction between the two therapies are similar. However, the studies included in our meta-analysis are relatively small, we could not do a subgroup analysis according to follow up duration and diabetic types, so further large, multi-center, high-quality studies are necessary to investigate.

## Ethical considerations

Ethical issues (Including plagiarism, informed consent, misconduct, data fabrication and/or falsification, double publication and/or submission, redundancy, etc.) have been completely observed by the authors.
